# Research on Efficient Reinforcement Learning for Adaptive Frequency-Agility Radar

**DOI:** 10.3390/s21237931

**Published:** 2021-11-27

**Authors:** Xinzhi Li, Shengbo Dong

**Affiliations:** Beijing Institute of Remote Sensing Equipment, Beijing 100854, China; shbdong@aliyun.com

**Keywords:** radar anti-jamming, reinforcement learning (RL), frequency-agility radar, Markov decision process (MDP), Markov game (MG)

## Abstract

Modern radar jamming scenarios are complex and changeable. In order to improve the adaptability of frequency-agile radar under complex environmental conditions, reinforcement learning (RL) is introduced into the radar anti-jamming research. There are two aspects of the radar system that do not obey with the Markov decision process (MDP), which is the basic theory of RL: Firstly, the radar cannot confirm the interference rules of the jammer in advance, resulting in unclear environmental boundaries; secondly, the radar has frequency-agility characteristics, which does not meet the sequence change requirements of the MDP. As the existing RL algorithm is directly applied to the radar system, there would be problems, such as low sample utilization rate, poor computational efficiency and large error oscillation amplitude. In this paper, an adaptive frequency agile radar anti-jamming efficient RL model is proposed. First, a radar-jammer system model based on Markov game (MG) established, and the Nash equilibrium point determined and set as a dynamic environment boundary. Subsequently, the state and behavioral structure of RL model is improved to be suitable for processing frequency-agile data. Experiments that our proposal effectively the anti-jamming performance and efficiency of frequency-agile radar.

## 1. Introduction

Frequency domain is an important field of radar electronic countermeasure (ECM). Because frequency agile radar has excellent anti-jamming performance and high range resolution, it is used in electronic countermeasures. With the advancement and progress of cognitive interference methods [1,2], the need to enhance the radar anti-jamming performance has increased. Frequency agile radar needs to have the ability to “intelligently” [3] change the radar frequency and adaptively select countermeasures [4] according to the tasks performed in the actual working environment. The design of the existing anti-jamming processing strategy relies on expert experience, and the working mode unable to change along with the environment and the target. When confronted with complex interference scenarios, artificially designed anti-jamming frequency-agility methods become cumbersome and difficult to implement, resulting in a decrease in radar anti-jamming performance. Therefore, it is an inevitable trend for frequency agile radar to have environmental perception and intelligent anti-jamming capabilities [5].

After Simon Haykin [6] first proposed the concept of cognitive radar in 2006, brain science [7] and artificial intelligence [8] were introduced into the radar system to adapt it to the complex and changing electromagnetic environment. Some studies [9,10] use support vector machine (SVM) [11] to identify and classify radar anti-jamming behavior to improve the anti-jamming effect of radar. Compared with traditional methods, this kind of methods improves the processing accuracy. Nevertheless, those methods depend on the prior knowledge to select the kernel function. In 2018, Liu et al. [12] proposed the use of deep learning (DL) [13] algorithm to evaluate the environment based on sample data, which was used as a basis for decision-making and achieved good results. However, methods based on deep learning rely on a large number of sample data, and cannot obtain good generalization performance in scenarios, e.g., incomplete information games. In 2017, Deligiannis et al. [14] adopted a Bayesian game theory framework to maximize the signal-to-interference-plus-noise ratio (SINR) between the radar and the target, and verified the convergence of the algorithm model through simulation results. In 2020, Garnaev et al. [15] used the Bayesian game model to construct an incomplete information relationship between the radar and the jammer, and optimized the performance of communication and radar components. Although the Bayesian game model does not need to know the objective function of the agent, it needs to master the distribution function and determine the optimal strategy through a specific probability. In 2010, Li et al. [16] tried to use RL [17] algorithm in cognitive radio countermeasures. After that, reinforcement learning (RL) algorithms were introduced and applied to radar systems to enhance radar anti-jamming performance. For example, in 2017, Qiang et al. [18] used the classic RL Q-learning (QL) algorithm [19] to make optimal allocation decisions for radar transmit power. This algorithm quantifies the performance of radar anti-jamming decision-making by establishing QTable [20] to record the state of the model. In 2019, Li et al. [21] proposed a deep Q-network (DQN) [22] based frequency agile radar strategy design method, which uses a neural network to approximate the anti-interference decision value function to guide the radar to select the optimal pulse carrier frequency. In 2019, Aref et al. [23] used the improved Double Deep Q-network (DDQN) [24] to conduct autonomous anti-jamming research in the radar frequency domain, and solved the problem of local anti-jamming optimal decision-making. In 2020, Ak et al. [25] studied the cognitive radar anti-jamming technology under the POMDP model, and quantified the performance of the radar being jammed through the conventional signal-to-noise ratio (SNR). Then use DQN and long-short term memory (LSTM) [26] network to find the optimal solution for the two frequency hopping strategies of the radar, which improves the anti-jamming performance of the radar.

RL is an interactive trial-and-error learning method, which can be applied to real-time update scenarios, e.g., online learning [27]. Through continuous trial and error, rewards from the environment can be used to continuously adjust one’s own behavior without requirement for a large amount of prior knowledge of the environment. This is an online learning method that can be applied to the actual environment, so it has been extensively studied [28,29,30]. However, existing RL algorithms rely on certain prerequisites, that is, the agent can conduct sufficient experiments in the environment, which is the experimental environment is Markov decision process (MDP) [31]. In this case, RL can ensure the convergence of the optimal strategy. The radar system does not satisfy MDP in two aspects: on one hand, the system contains multiple agents, e.g., radar and jammers, which is beyond the scope of application of RL based on MDP and violates the assumption of algorithm convergence. On the other hand, the decision action taken by the radar is not a typical sequence decision process [32], and there is the possibility of step changes. That is, from the current parameter action with weak anti-interference ability to the parameter action with best anti-interference ability, the relationship between each action is independent. It is also not satisfied with MDP. Therefore, it is difficult for algorithms based on existing RL models to obtain the correct evaluation of current strategies from the radar system environment. This will lead to poor convergence and low computational efficiency of the algorithm in the environment, which cannot meet the high-precision and high-real-time requirements.

In conclusion, existing intelligent algorithms cannot be directly applied to radar systems: both SVM and DL algorithms require a great quantity of prior knowledge and do not have online learning capabilities; the Bayesian game theory model relies on the distribution function; RL online learning can obtain global optimal decisions, but basic theoretical models and frameworks are difficult to apply to radar systems. In response to the above problems, combined with the characteristics of frequency-agility of frequency agile radar, this paper proposes an efficient and adaptive RL anti-jamming model for frequency-agile radar. Firstly, the Markov game (MG) [33] is introduced, which transforms the radar-jammer detection model into a reinforcement learning model that can satisfy the MG theory. Secondly, the state and behavioral framework structure of the original RL model is improved, so that the new framework structure can be applied to the frequency agile data, and the convergence of the framework is demonstrated through the Bellman equation. In the experiment, based on the frequency agile radar system environment, this paper compared the classical RL algorithms in the ideal MDP framework (ideal model, IM) and the improved framework (AM) proposed in this paper. According to the quantitative analysis of mean square error (MSE) and convergence speed, compared with the basic RL model, the algorithm model proposed in this paper can improve the anti-jamming performance and processing efficiency of frequency agile radar.

## 2. Reinforcement Learning Modeling Based on Radar System

### 2.1. Radar Countermeasure System Model

**Figure 1:**
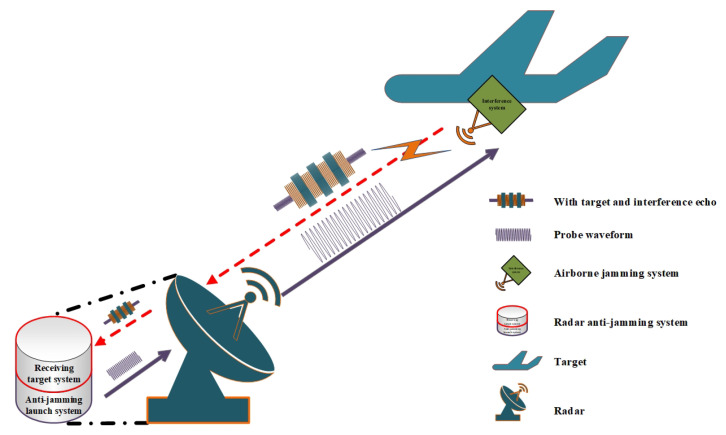
Anti-jamming principle diagram based on frequency agile radar radar and airborne jamming platform.

The environment of modern radar system usually contains a complex interference environment, which prevents the detection radar from obtaining accurate target information. As shown in Figure 1, the system model consists of two agents (frequency agile radar for detecting targets and airborne jammer): the radar obtains the echo signal through a receiver, and evaluates the transmit detection signal through the effectiveness evaluation. Then, it makes a decision and launchs a new anti-jamming detection signal. The jammer receives the radar detection signal and performs real-time interference processing.

**Figure 2:**
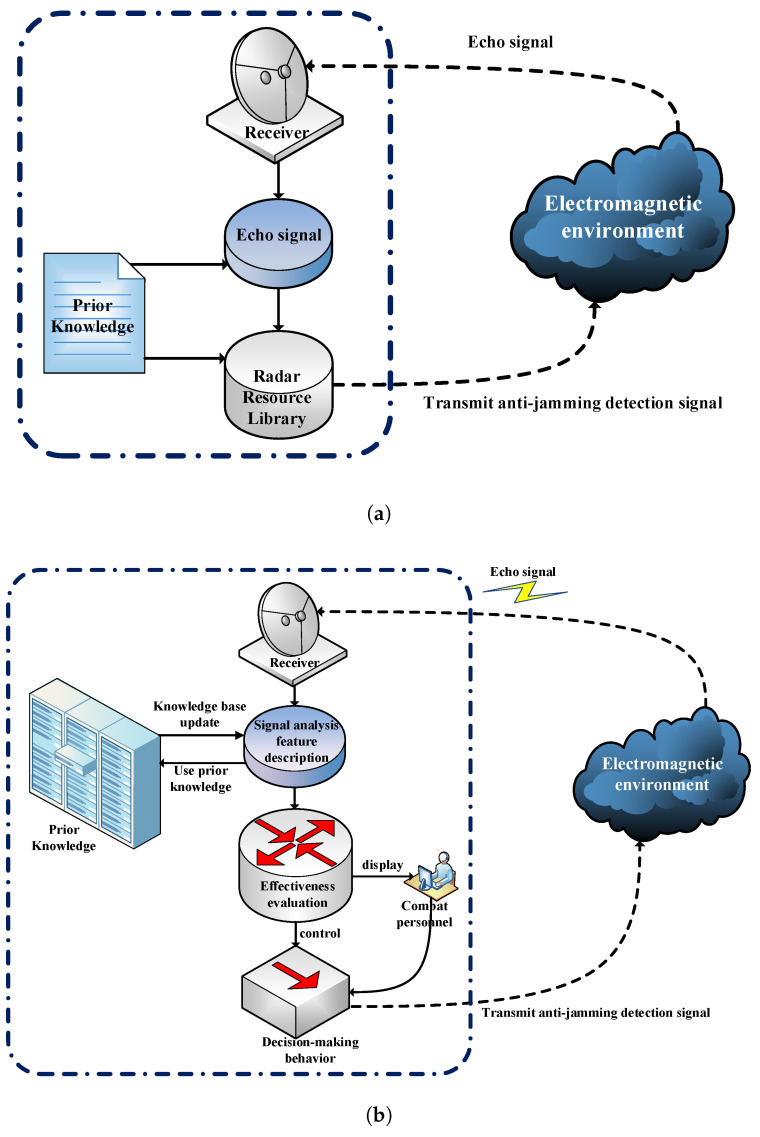
Comparison chart of radar system anti-jamming process. (**a**) Traditional radar anti-jamming model, (**b**) Intelligent radar anti-jamming model.

Traditional frequency agile radar usually adopts “cover pulse + tracking signal” composite detection waveform [34,35] to achieve anti-jamming measures. That is, the radar transmitter transmits two kinds of signals. One is a cover pulse with obvious spectral characteristics, which is used to guide the jammer to lock the frequency and waveform of the jamming signal to the cover pulse. The other is a tracking signal with low interception characteristics for real detection and tracking functions. The tracking signal gets staggered with the cover pulse and reduces the effective power of the jamming signal into the radar machine, so as to achieve the anti-jamming effect. The traditional radar anti-jamming system is shown in Figure 2a. First, the radar receiver receives echo signals from the electromagnetic environment to obtain specific measurement results of target and interference signal parameters. Then, based on the detected and existing prior knowledge base, e.g., pulse width, modulation method and repetition period of the target signal and the interference signal, which are used to determine the type of the interference signal. Finally, combine the target and interference signal measurement results to schedule anti-interference resources, select effective anti-interference cover pulse and tracking signal, and transmit them through the transmitter.

**Figure 3:**
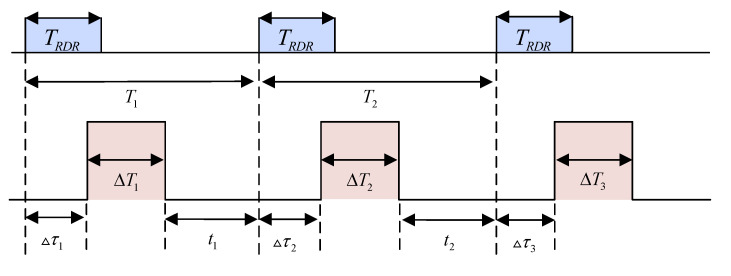
Synchronous aiming jamming timing diagram.

According to the anti-interference characteristics of frequency agile radar, jammers usually use synchronous targeting jamming [36,37] in suppressive jamming for signal jamming. As shown in Figure 3, the jammer performs frequency targeting on each radar pulse received. Then, it interferes with the current radar signal and stops after $\Delta T_{1}$ time. After the next pulse arrives, the jammer will perform frequency aiming again, and stop after jamming for a period of time $\Delta T_{2}$, and so on.

In Figure 3, the pulse repetition period of the $T_{1}$ and $T_{2}$ frequency agile radar. The distance between the radar and the target is determined by the propagation speed c and the waveform propagation time $T_{RDR}$. Here, $T_{RDR}$ is used to describe the distance between the target and the radar. $\Delta \tau_{1}$, $\Delta \tau_{2}$ and $\Delta \tau_{3}$ are the delay time of each pulse aiming frequency. $\Delta T_{1}$, $\Delta T_{2}$ and $\Delta T_{3}$ are the real-time jammers based on the aiming results Interference time; $t_{1}$ and $t_{2}$ are the stop interference time.

Synchronous targeting jamming technology can effectively interfere with frequency agile radar. Moreover, it is irrelevant to the frequency agile modulation method. When the frequency agile radar system detects that the interference signal parameters do not exist in the prior expert knowledge, it cannot dynamically adjust the anti-jamming detection radar signal pattern based on the anti-jamming effect feedback obtained from the environment. In this situation, the anti-jamming effect of radar deteriorates drastically, and the processing efficiency reduces greatly.

Compared with traditional radar system relying on a static prior knowledge base to make decisions, intelligent radar anti-jamming system has the ability to dynamically perceive the external environment and optimize the knowledge base. The system can dynamically adjust the anti-jamming measures based on the feedback of the anti-jamming measures from the external environment (whether the target is tracked, whether the jamming signal is locked to the shield pulse). The intelligent radar anti-jamming system is shown in Figure 2b. First, the echo signal is received from the receiver, and the specific measurement results of the target and interference signal parameters are obtained. Then, the interference pattern is analyzed according to the prior knowledge base. In this case, if the interference pattern does not exist in the prior knowledge base, it is necessary to identify and analyze the situation of the target and the interference signal, and evaluate the current anti-jamming effectiveness. The evaluation content includes the accuracy evaluation of the target information and whether the jamming signal is locked to the shielding pulse, etc. Finally, the anti-jamming method is adjusted according to the results of the effectiveness evaluation and launched. In addition, add the newly detected interference pattern feature parameter information and effectiveness evaluation results into the prior knowledge base. The intelligent radar anti-jamming system continuously interacts with the external environment to learn online anti-jamming strategies that are not in the prior knowledge base. The system can finally form a set of optimal strategies for a specific environment based on the accumulation of experience.

Contrapose different radar system environments in the intelligent radar anti-jamming system, we have established a RL model suitable for a single radar system and a RL model suitable for a radar-jammer system environment. We analyze the reasons why the existing basic theoretical models and frameworks of RL are not suitable for the environment of radar-jammer system, and propose an efficient RL model suitable for the system.

### 2.2. Reinforcement Learning Model for Single Radar System

**Figure 4:**
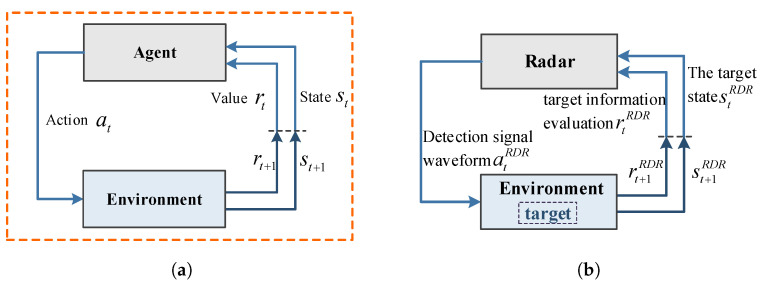
Comparison chart of basic RL theory model and single radar system model. (**a**) Basic reinforcement learning model framework, (**b**) Intelligent radar anti-jamming model.

MDP is the theoretical derivation basis of single-agent RL [38], and solving RL problems depends on the framework. As shown in Figure 4a, the Markov decision process is a tuple <s,a,t,r> composed of four elements, where *s* represents the limited set of all states contained in the environment by the agent, a represents the limited set of all actions that the current agent will take in the environment, *T* represents the transformation equation, and *r* is the reward equation. The decision process sequence of the agent under the MDP framework can be expressed as { $s_{0},a_{0},r_{1},s_{1},a_{1},r_{2},s_{2},a_{2},r_{3},...$ }, where $r_{t}$ and $s_{t+1}$ only depend on the previous state $s_{t}$ and action $a_{t}$, and *t* represents time.

The frequency agile radar system based on the RL theory first launches the frequency agile detection signal source strategy $a_{t}^{RDR}$. Then the echo signal is received by the receiver. Finally, according to the current target state $s_{t}^{RDR}$ and the detection result evaluation $r_{t}^{RDR}$, select and transmit the next anti-interference agile detection signal source strategy $a_{t+1}^{RDR}$. The radar system obtains target status information by continuously interacting with the environment. According to the evaluation result of the target status information, the transmitted frequency signal is changed to improve the radar’s anti-jamming detection target ability in real time.

Early radar systems were simple and usually contained only a radar and a target. As shown in Figure 4b, the simple single radar-target environment system satisfies the MDP condition, which can be analogous to the RL theoretical model. The radar in the radar system which is regarded as an agent, and together with the target and its environment, it is regarded as a complete MDP environment. Its parameters are defined as shown in Table 1. During the interaction between the radar and the environment, the goal of the radar is to adjust the emitted signal source to maximize the detection of the target’s position, speed and acceleration in the environment.

This paper gives the parameter definition of RL model based on single radar system: Assuming a discrete time sequence t=0,1,2,3..., at each time *t*, the radar can detect the state $s_{t}^{RDR}$ of a target from the environment. Define the signal source taken by the radar at time *t* as action $a_{t}^{RDR}$. At the next moment, $s_{t+1}^{RDR}$ is defined as the received value return $r_{t+1}^{RDR}$ as the result of the radar taking action $a_{t}^{RDR}$ to evaluate whether the detected target is accurate. At the next moment, $s_{t+1}^{RDR}$ defines the result of the radar taking action $a_{t}^{RDR}$ in the current state $s_{t}^{RDR}$, and assessing whether the detected target is accurate and other information is defined as taking action $a_{t}^{RDR}$ to obtain value return $r_{t+1}^{RDR}$. At each moment, the radar completes the mapping from the state $s_{t}^{RDR}$ to the selection probability of each possible signal action $a_{t}^{RDR}$. This mapping relationship becomes the radar strategy, denoted as $\pi_{t}^{RDR}$, $\pi_{s,a}^{RDR}$ which is the probability of selecting $a_{t}^{RDR}$ when the state is $s_{t}^{RDR}$.

Through the above analysis, a single radar system can be analogous to a RL theoretical model. However, when there are multiple agents in the radar system environment (radar, jammer, and target), the MDP-based RL model framework cannot be directly applied. It is necessary to introduce new theories and conditional constraints to ensure the convergence of the algorithm model.

### 2.3. Reinforcement Learning Modeling for Radar-Jammer System

With the rapid development of information technology and its widespread application in the military field, the application environment of modern radars has become increasingly complex. Usually, the combat environment contains radar and jammer, and there is an interference effect between the two to prevent the detection radar from obtaining accurate information about the target. In the above scenario, the existing RL algorithm model cannot be directly applied to the radar-jammer countermeasure system. RL is developed based on the MDP theory. It is a strategy for a single agent to learn the possible delayed return signal maximization strategy in a random static environment, e.g., QL [18], DQN [21], DDQN [24], DQN+LSTM [25] and other classic algorithm models. This type of algorithm relies on certain prerequisites, that is, the agent can perform sufficient experiments in the environment, and the experimental environment is MDP, then RL can ensure the convergence of the optimal strategy.

The radar-jammer system does not satisfy the MDP theory in two aspects: On one hand, the rader-jammer system is beyond the scope of MDP theoretical application. The main reason is that in this kind of environmental system, the optimal strategy of the agent not only depends on the environment, but also on the strategies adopted by other agents, which violates the assumptions required to ensure convergence. When agents have opposing targets, there may not be a clear optimal strategy, resulting in frequent retrieval of the balance between multiple agents, making the environment unstable. On the other hand, the decision-making action taken by the radar is not a typical sequence decision-making process, and there is the possibility of step changes. That is, a step from the current parameter action with weak anti-interference to the parameter action with good anti-interference, and the relationship between each action is independent, which also does not satisfy the MDP theory. Therefore, in the complex electromagnetic environment of the radar system with multiple agents coexisting, it is difficult for the existing RL algorithm to obtain the correct evaluation of the current strategy from the environment, resulting in poor algorithm convergence and unable to satisfy the real-time requirements of military radars.

**Figure 5:**
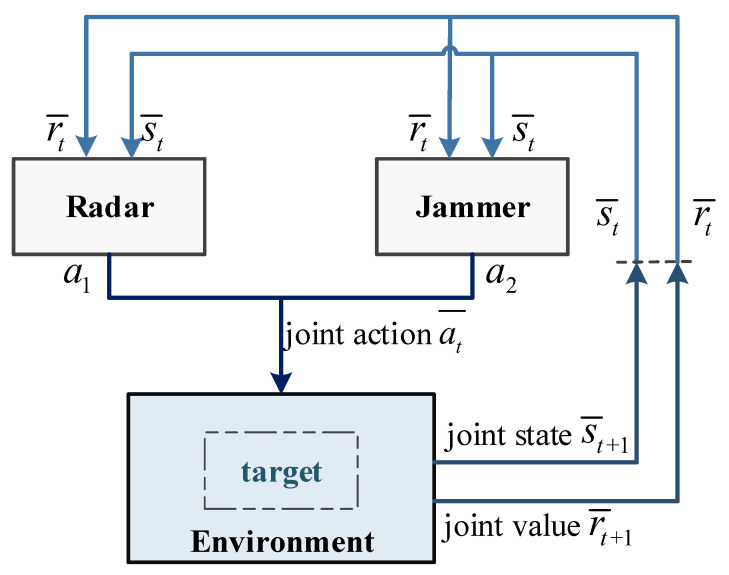
Environment model of radar system based on MARL.

The combination of interdisciplinary game theory and artificial intelligence RL is the theoretical basis of solutions in the field of multi-agent reinforcement learning (MARL) [31]. The characteristic of a multi-agent system is the interaction of strategies between multiple agents. Each agent has independent goals and decision-making capabilities, and at the same time, the agent is affected by the behavioral decisions of other agents. When the radar is in a multi-agent environment, the value return of the radar is affected by the behavioral decisions of the jammer. According to MG theory, the radar and the jammer independently choose actions to form a joint action. If the learning model of multi-agents in the same environment is adopted, the process is shown in Figure 5, e.g., the actions $a_{1}$ and $a_{2}$ taken by the radar and the jammer are regarded as shared actions, and joint actions $\bar{a_{t}}$ are adopted to obtain joint state $\bar{s_{t}}$ and value return $\bar{r_{t}}$.

**Figure 6:**
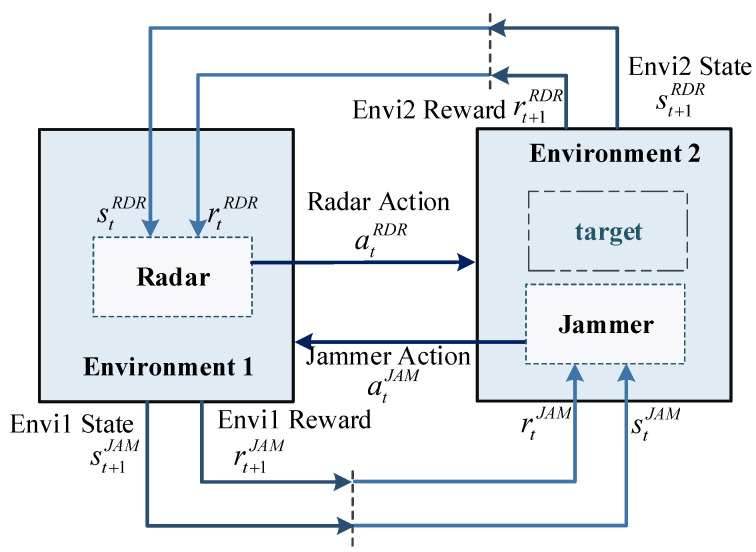
The environment model of radar-jammer system proposed in this paper.

However, in reality, the radar and the jammer cannot know the status and action strategy of the other side, and should regard the other side as part of the environment. As shown in Figure 6, this paper proposes a radar-jammer system environment model, which regards the radar and the jammer as two groups of agent environments: radar is environment1 (Envi1), jammer and target are composed of environment2 (Envi2).

The parameter definitions of the radar side and the jammer are shown in Table 2. For the radar side, the environment of the jammer and the target is regarded as Envi2. The radar sends a signal $a_{t}^{RDR}$ to Envi2, from which a target state $s_{t}^{RDR}$ with jamming information and a return value $r_{t}^{RDR}$ to evaluate the accuracy of the target are obtained. Then the transmitting signal behavior $a_{t}^{RDR}$ of the radar is adjusted to detect the accurate target information.

Where, the target error rate $\rho_{d}$ refers to the false target probability that the target in the echo signal detected by the radar front-end detector is the jammer. The jammer uses the adaptive radar to interfere with the synchronized aiming jamming technology in Section 2.1. Nevertheless, it cannot optimize interference strategies based on environmental feedback.

This section proposes a radar-jammer system environment model suitable for the actual radar system environment. The theoretical basis of this model will be analyzed in Section 2.4.

### 2.4. Markov Game Inference Applicable to Radar-Jammer System Environment

MDP includes one agent and multiple states. Radar-jammer system is a typical special case of multi-agent systems, and MDP theory cannot be applied to the multi-agent systems. For a game with multiple agents and multiple states, Markov game (MG) is defined. MG uses a tuple to represent { $n,s,a_{1},\ldots ,a_{n},T,t_{1},\ldots ,t_{n}$ } where *n* represents the number of multi-agent in the current environment, *T* represents the transfer function, $a_{i}$ (i=1,...,n) represents the behavior set of the *i*th agent, and $r_{i}$ represents the reward function of the agent *i*. The transfer function *T* in MG refers to the probability distribution of the next state when the current state and joint behavior of the agent are given. The reward function $r_{i}$ ($s,a_{1},...,a_{n},s^{\prime }$) indicates that the agent *i* takes the joint in the state *s*. After the behavior ($a_{1},...,a_{n}$), the reward is obtained in the next state $s^{\prime }$.

Combining with MG, give the definition of a radar-jammer countermeasure system. Assuming that the system contains a radar and a jammer, a tuple can be expressed as {$2,s^{RDR},a^{RDR},a^{JAM},T,r^{RDR},r^{JAM}$}:

1. 2 refers to the number of agents in the radar-jammer system, including the radar and the jammer;
2. *s* = {$s^{RDR},s^{JAM}$} indicates the joint state of the radar-jammer system model, $s^{RDR}$ indicates the current state of the radar side, and $s^{JAM}$ indicates the state of the jammer;
3. $a^{RDR}$ represents the action set of the radar, and $a^{JAM}$ represents the action set of the jammer;
4. *T* represents the transfer function, which represents the probability of the radar side taking action $a^{RDR}$ and the jammer taking action $a^{JAM}$ under the current state and then transferring to the next state;
5. $r^{RDR}$ represents the reward function of radar; $r^{JAM}$ represents the reward function of jammer. Among them, the radar side and the jammer side have independent reward function *R*. Different agents can switch from the same state to different rewards.

For judging whether the MG is applicable to the model, the focus is to determine whether there is a Nash equilibrium in the model. The core of game theory is to establish a strategy interaction model for the game between multi-agent. Each game is equivalent to a mathematical model, which is used to describe the interaction results of each agent’s reward strategy. Combined with a radar-jammer system, the process of target detection and recognition by the radar each time is a game. Each target detection is performed by selecting a group of signals $a^{RDR}$ from the set of transmitted radar waveform signals (as actions $a^{RDR}$) by the radar.

Radar-jammer systems are different from games, e.g., Go [21]: the mutual gains of the radar and the jammer will become part of the other party’s losses. However, in the actual situation, the two parties are not sure that they will bring losses to the other party, which is a general game and there is a Nash equilibrium. As shown in Table 3, taking two actions each of the two agents as an example, analyze the Nash equilibrium between the detection target radar and the jammer: The vertical radar side takes actions $a_{i}^{RDR}$ (i=1,2), and the horizontal jammer side takes actions $a_{i}^{JAM}$ (i=1,2). The two have their own optimal decision-making actions $a_{i}^{RDR}$ and $a_{i}^{JAM}$, respectively.

Combining the Nash equilibrium theorem, as shown in Equation (1), set $\pi =(\pi_{RDR},$$\pi_{JAM})$ to be the joint strategy of radar-jammer system, $\pi_{RDR}$ to indicate the radar strategy, and $\pi_{JAM}$ to indicate the jammer strategy.

(1) $$r_{RDR}\left(\pi_{JAM},\pi_{RDR}^{*}\right)\geq r_{RDR}\left(\pi_{JAM},\pi_{RDR}^{\prime }\right)\forall \pi_{RDR}^{\prime }\in \mu \left(a^{RDR}\right),$$

where $\pi_{RDR}^{*}$ represents the strategy implemented by the jammer in the system except for the radar side, $r_{RDR}$ represents the radar return function, $\mu (a^{RDR})$ represents the action set of the radar, and represents the probability distribution set on the action set $a^{RDR}$ of the radar. When Equation (1) holds, it means that strategy $\pi_{RDR}^{*}\in \mu \left(a^{RDR}\right)$ is the optimal strategy of the radar side. Here $\left(\pi_{JAM},\pi_{RDR}^{\prime }\right)$ indicates that when the jamming strategy $\pi_{JAM}$ is fixed, the radar adopts an arbitrary strategy $\forall \pi_{RDR}^{\prime }\in \mu \left(a^{RDR}\right)$. $\left(\pi_{JAM},\pi_{RDR}^{*}\right)$ means that when the jamming strategy $\pi_{JAM}$ is fixed, the strategy $\pi_{RDR}^{*}$ adopted by the radar is the optimal strategy. In the game process, when the radar side makes the optimal decision, the interference side keeps its strategy unchanged, the current radar side cannot further improve its return, and the Nash equilibrium is reached at this time. According to this, we give the definition of the Nash equilibrium of the radar-jammer system: in the game model $G=\{\pi_{RDR},\pi_{JAM}$: $\mu \left(a^{RDR}\right),\mu (a^{JAM}$)}, if the strategy combination of radar and jamming ($\pi_{RDR}^{*},\pi_{JAM}^{*}$), the strategy $\pi_{i}^{*}$ (i=RDR,JAM) of either party is the best strategy for the other party’s strategy $\pi_{j}^{*}$ (j≠i). It is said that ($\pi_{RDR}^{*},\pi_{JAM}^{*}$) is a Nash equilibrium of the radar-jammer system. Because the relationship between radar and jammer is confrontation, the two parties cannot adopt the optimal strategy at the same time. Therefore, the ($a_{2}^{RDR}$, $a_{2}^{JAM}$) in Table 3 does not exist in the radar-jamming system. Regarding this point, we set ($a_{2}^{RDR}$, $a_{1}^{JAM}$) to be the Nash equilibrium state of the radar’s successful anti-interference, ($a_{1}^{RDR}$, $a_{2}^{JAM}$) is the Nash equilibrium state of the jammer’s successful interference.In the process of the game between the radar and the jammer, there exists a Nash equilibrium of one side’s optimal strategy. At this time, the MG theory can be used in the radar-jamming system environment model proposed in this paper.

## 3. An Improved RL Framework for Radar-Jammer System

**Figure 7:**
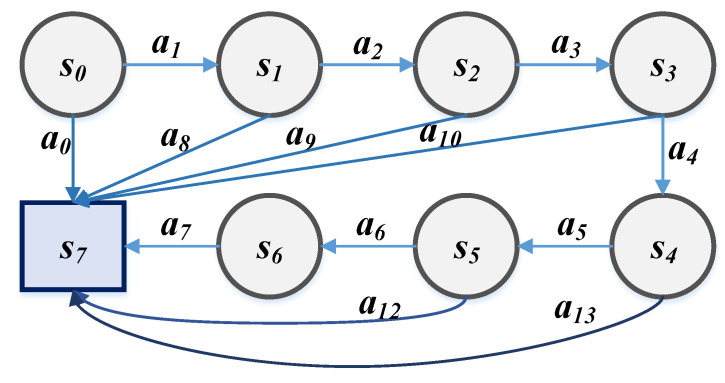
Radar system state-action transition diagram.

The frequency signal of frequency agile radar changes in steps rather than continuously. As shown in Figure 7, the jump target of the radar is to take action $a_{0}$ from initial state $s_{0}$ to final state $s_{7}$ where the definition of radar state $s_{i}$ (i=1,...,7) is shown in Table 4. When the radar takes different actions $a_{i}$ (i=0,1,2,...,13), it will jump from the current state *s* to other states s’ with a certain probability. However, the difference from the Markov chain is that certain existing states and behaviors are not necessarily paths, as shown by the six paths in Equations (2)–(7). Because in the radar system environment, the echo signal is affected by the interference signal. At this point, according to the complete Markov decision theory, it can be obtained from the value echo *r* of the environment that taking behavior $a_{0}$ in the $s_{0}$ state is the best strategy. However, the radar-jammer system environment is based on the MG theory, and the radar and the jammer affect the behavior strategy. It is difficult for the radar side to implement behavior $a_{0}$.

(2) $$s_{0}\overset{a_{1}}{\to }s_{1}\overset{a_{2}}{\to }s_{2}\overset{a_{3}}{\to }s_{3}\overset{a_{4}}{\to }s_{4}\overset{a_{5}}{\to }s_{5}\overset{a_{6}}{\to }s_{6}\overset{a_{7}}{\to }s_{7}$$

(3) $$s_{0}\overset{a_{1}}{\to }s_{1}\overset{a_{2}}{\to }s_{2}\overset{a_{3}}{\to }s_{3}\overset{a_{4}}{\to }s_{4}\overset{a_{5}}{\to }s_{5}\overset{a_{12}}{\to }s_{7}$$

(4) $$s_{0}\overset{a_{1}}{\to }s_{1}\overset{a_{2}}{\to }s_{2}\overset{a_{3}}{\to }s_{3}\overset{a_{4}}{\to }s_{4}\overset{a_{13}}{\to }s_{7}$$

(5) $$s_{0}\overset{a_{1}}{\to }s_{1}\overset{a_{2}}{\to }s_{2}\overset{a_{3}}{\to }s_{3}\overset{a_{10}}{\to }s_{7}$$

(6) $$s_{0}\overset{a_{1}}{\to }s_{1}\overset{a_{2}}{\to }s_{2}\overset{a_{9}}{\to }s_{7}$$

(7) $$s_{0}\overset{a_{1}}{\to }s_{1}\overset{a_{8}}{\to }s_{7}$$

For the above problems, this paper improves from two aspects. Firstly, the state and behavior frame structure of the original RL model is improved to make the new frame structure suitable for the frequency agile data. Then, it is proved that the state value function satisfies the Bellman equation in the frame structure.

### 3.1. Improved RL Framework

**Figure 8:**
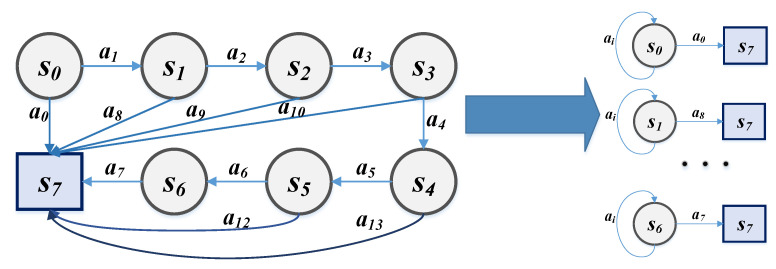
Suitable for frequency agile scenario state-action transition diagram.

Value evaluation is a method used to connect optimal criteria and strategies in RL. The agent needs to calculate the optimal strategy based on the value evaluation obtained from the environment feedback. In the application of RL based on radar system, J/R [39,40] is usually used to evaluate the value of the state. At this time, the following two problems exist in the continuous state of RL to build the model. On one hand, in the radar-jammer system environment, the jammer, which making the radar status and the strategy adopted cannot be directly correlated, affects the radar status and the status is difficult to quantitatively evaluate. On the other hand, the radar signal changes step by step and needs to be evaluated based on the radar echo signal, which has time-delay. To solve the above problems, the state transition model of RL is improved in this paper. Sets the echo signal received by the radar to state *s*, removes the intermediate state, and only retains the initial state and the final state. As shown in Figure 8, in the initial state $s_{0}$ of the radar, the final state $s_{7}$ is reached only when the action $a_{0}$ is taken, and the initial state $s_{0}$ is reached when other actions $a_{i}$ (i=1,2,...,13) are taken; each state generated in the intermediate process is regarded as the current initial state $s_{0}$.

The proposed method can effectively reduce the influence of the jammer and avoid the strategic association with the irrelevant state of the middle part. It is suitable for radar frequency-agility scenarios. This framework satisfies the MDP, reduces the data noise interference contained in the intermediate state, effectively enhances the robustness of the model, and greatly improves the training efficiency and inference performance of the model.

### 3.2. Bellman Equation in Frequency-Agility Scenario

The foundation and core of RL is Bellman equation. In the process of obtaining the optimal strategy, the fundamental goal of RL is to optimize the strategy function π(s), thereby maximizing the value function. The state value function is one of the criteria for evaluating the quality of the strategy function. In the basic model of RL, each state *s* (s∈S, where *S* is the set of all states) can have multiple choices of action *a* (a∈A where *A* is the set of all actions). When action *a* is performed, the system will transition to another state s’ according to a certain probability $P_{ss^{\prime }}^{a}$. At this point, in this framework, each state *s* is related through action *a*, and there is a connection between the states. Under this precondition, the state value function can effectively evaluate the strategy function and form a recursive form, as shown in the following Equation (8):

(8) $$\begin{array}{cc} V^{\pi }\left(s\right) & =E^{\pi }\left[R_{t}|s_{t}=s\right]\\ \; & =E^{\pi }\left\{\sum_{k=0}^{\infty }\gamma^{k}r_{t+k+1}|s_{t}=s\right\}\\ \; & =E^{\pi }\left\{r_{t+1}+\gamma \sum_{k=0}^{\infty }\gamma^{k}r_{t+k+2}|s_{t}=s\right\}\\ \; & =\sum_{a}\pi \left(s,a\right)\sum_{s^{\prime }}P_{ss^{\prime }}^{a}\left[R_{ss^{\prime }}^{a}+\gamma E^{\pi }\left\{\sum_{k=0}^{\infty }\gamma^{k}r_{t+k+2}|s_{t+1}=s^{\prime }\right\}\right]\\ \; & =\sum_{a}\pi \left(s,a\right)\sum_{s^{\prime }}P_{ss^{\prime }}^{a}\left[R_{ss^{\prime }}^{a}+\gamma V^{\pi }\left(s^{\prime }\right)\right], \end{array}$$

where, $V^{\pi }\left(s\right)$ represents the state value function, and $P_{ss^{\prime }}^{a}$ represents the probability of state *s* transitioning to state $s^{\prime }$ when action a is selected. $R_{ss^{\prime }}^{a}$ represents the reward for the transition from state *s* to next state $s^{\prime }$ when action *a* is selected, which is replaced by $R_{t}$ and expressed by the following Equation (9):

(9) $$\begin{array}{c} R_{t}=\sum_{k=0}^{\infty }\gamma^{k}r_{t+k+1}, \end{array}$$

where, $r_{t+1}$ represents the value return from state *s* to state $s^{\prime }$, and γ is the loss factor, value range is (0,1). $R_{t}$ determines the probability distribution at the end of each round of training.

The RL framework based on the radar system environment proposed in this paper also satisfies the state value function of the Bellman equation: Set γ=1 when the initial state *s* is transferred to the final state $s^{\prime }$ by taking action *a*. In other cases γ=0. This situation is equivalent to the special equation solution of Bellman equation. As shown in the following Equation (10):

(10) $$\begin{array}{c} V^{\pi }\left(s\right)=\left\{\begin{array}{ccc} \sum_{a}\pi \left(s,a\right)\sum_{s^{\prime }}P_{ss^{\prime }}^{a}\times R_{ss^{\prime }}^{a}, & s\to s & \\ \sum_{a}\pi \left(s,a\right)\sum_{s^{\prime }}P_{ss^{\prime }}^{a}\left[R_{ss^{\prime }}^{a}+V^{\pi }\left(s^{\prime }\right)\right], & s\to s^{\prime } \end{array}\right. \end{array}$$

According to theoretical analysis, the model framework proposed in this paper is a special equation solution of Bellman equation, which demonstrates the effectiveness of the enhanced model under the radar system environment. In this framework model, the radar system model that satisfies the MG theory is converted into an environment model that satisfies the MDP, which ensures the convergence of the algorithm.

## 4. Experiments

### 4.1. Setup

In order to verify the availability of the proposed model in the radar-jammer system environment, the radar side and the jammer side simulation environment models were built on the TensorFlow platform. The four algorithms QL [18], DQN [21], DDQN [24], DQN+LSTM [25] are compared in the IM (Ideal Model, IM) and the proposed AM (Advanced Model, AM). The four RL methods are briefly described as follows:

- QL (Q-learning) [18]: Q-learning (QL) is a classic reinforcement learning algorithm, which can efficiently uses guided experience replay sampling to update the strategy, and selects the action that can get the most benefit based on the Q value.
- DQN (deep Q-network) [21]: Deep Q-learning replaces the regular Q-table with a neural network. Rather than mapping a state-action pair to a q-value, a neural network maps input states to (action, Q-value) pairs. DQN algorithm relies on limited discrete empirical data storage memory and complete observation information, effectively solving the “dimension disaster” of reinforcement learning.
- DDQN (double deep Q-network) [24]: DDQN is an optimization of the DQN algorithm, using two independent Q-value estimators, each of which is used to update the other. Using these independent estimators, an unbiased Q-value estimation can be performed on actions selected using the opposite estimator. Therefore, the update can be separated from the biased estimate in this way to avoid maximizing bias.
- DQN+LSTM (deep Q-network + long-short term memory) [25]: DQN+LSTM is proposed to solve two problems of DQN: (1) The memory for storing empirical data is limited. (2) Need complete observation information. DQN+LSTM replaces the fully connected layer in DQN with an LSTM network. In the case of changes in observation quality, it has stronger adaptability.

To test the convergence of the algorithm, the experiment is set to 6000 rounds, including 8 effective radar states, of which $s_{7}$ is the effective final state. Radar behavior $a^{RDR}$ is regarded as a behavior group, which contains 14 behaviors. Each $a_{i}^{RDR}$ (*i* = 0,1,...,13) behavior sets a set of discrete reference values according to the real radar signal. The behavior of the jammer, $a^{JAM}$, was regarded as the behavior group, and 28 behaviors were selected. A set of discrete reference values was set for each behavior of $a_{i}^{JAM}$ (*i* = 0,1,...,27) according to the real jammer signals. The reward values of the radar side and the jammer are shown in Table 5. The reason for setting the reward value of the radar side and the jammer is as follows: because the radar side does not know the information of the jammer at the beginning of the confrontation, the initial value of the reward value is set to 0. In order to effectively improve the ability of radar online anti-jamming learning, high score rewards for effective anti-jamming measures and low scores for general anti-jamming measures are given based on environmental feedback. Since the jammer can interfere with the radar aiming frequency at the initial stage, the initial value is set to 30. However, this interference method cannot evaluate the interference effect based on the radar status to optimize the strategy. Therefore, the jammer is set to score according to the radar score, and the reward range in the positive and negative directions is less than that of the radar. The termination condition for each round is that the two parties first reach 200 points or the number of confrontations reaches 2000. Each experiment runs 100 times repeatedly, and the processing time was the average of 6000 rounds of experimental data.

### 4.2. Results and Analyss

#### 4.2.1. Convergence Analysis

**Figure 9:**
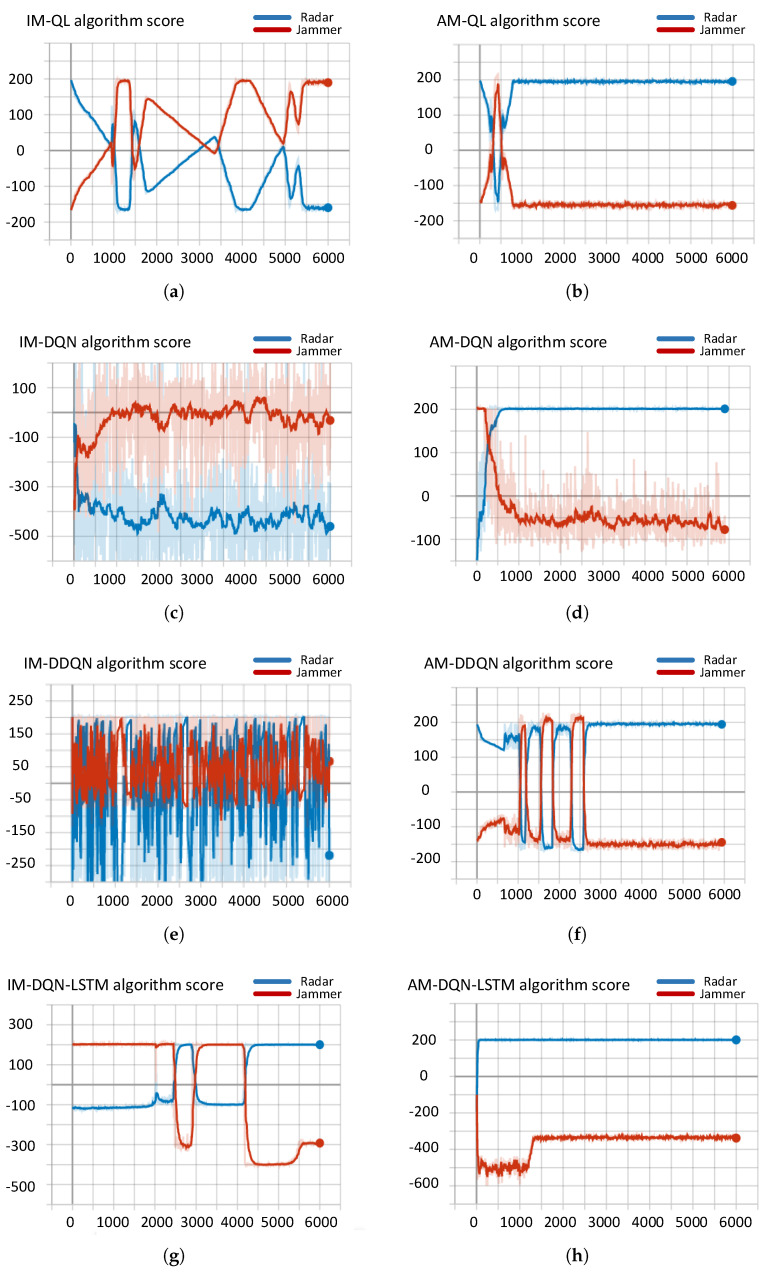
Comparisonof anti-jamming effects of classic RL algorithms under IM and AM models. (**a**) IM-QL, (**b**) AM-QL, (**c**) IM-DQN, (**d**) AM-DQN, (**e**) IM-DDQN, (**f**) AM-DDQN, (**g**) IM-DQN+LSTM, (**h**) AM-DQN+LSTM.

The experimental results are evaluated based on the anti-jamming effect of the radar. As shown in Figure 9, it is the experimental results of the radar and jammer countermeasures under IM and AM with four RL algorithms of QL, DQN, DDQN, and DQN+LSTM. In the figure, the horizontal axis represents the number of rounds, and the vertical axis represents the respective confrontation scores of the radar and the jammer. The red curve represents the jammer, and the blue curve represents the radar side.

It can be analyzed from the experimental results in Figure 9 that the classic RL algorithms QL and DQN under the IM model cannot achieve effective anti-jamming measures. The result is that the jammer wins the confrontation. In the DDQN and DQN+LSTM algorithms, the radar anti-jamming effect is unstable, and effective anti-jamming measures cannot be implemented. Under the AM framework, the same algorithms enable the frequency agile radar to quickly search the optimal anti-jamming strategy, implement effective interference countermeasures and achieve the ultimate victory. This experiment proves that the AM framework proposed in this paper can effectively solve the problem of the algorithm cannot converge in the radar-jammer system.

#### 4.2.2. Performance Analysis

**Figure 10:**
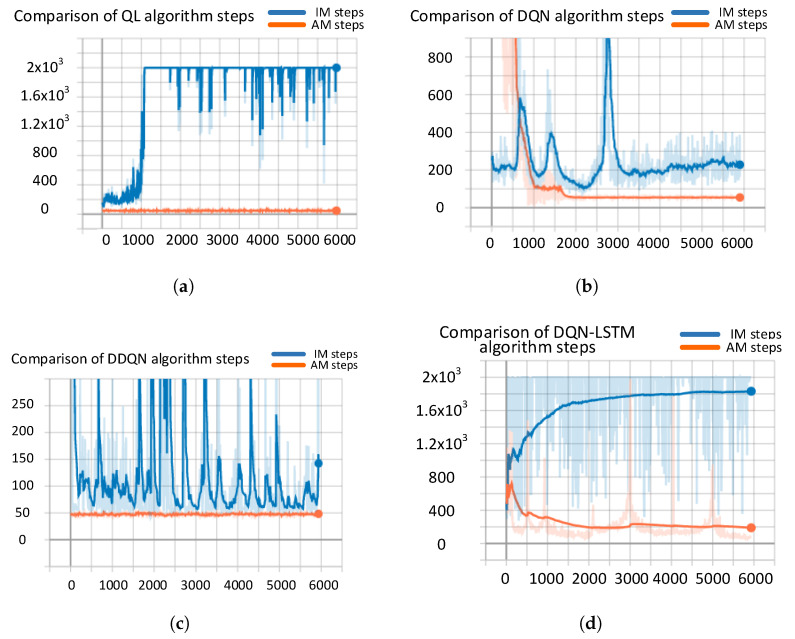
Convergence comparison chart of classical RL algorithms under IM and AM models. (**a**) QL algorithm comparison, (**b**) DQN algorithm comparison, (**c**) DDQN algorithm comparison, (**d**) DQN+LSTM algorithm comparison.

Figure 10 shows the number of confrontations per round between the radar side and the interferer under the four algorithms of QL, DQN, DDQN, and DQN+LSTM under IM and AM. The horizontal axis represents the number of rounds, and the vertical axis represents the number of confrontations per round. The blue curve represents the IM model, and the red curve represents the AM model.

Table 6 is the online processing time and cumulative mean square error (MSE) analysis of the four algorithms QL, DQN, DDQN, DQN+LSTM under IM and AM, respectively.

Among them, the convergence time refers to the selection strategy from the beginning to the stable selection strategy in a single round of the algorithm. The single-round decision time after convergence refers to the running time from each turn to the optimal strategy after stable strategy selection. The cumulative mean square error analysis is the cumulative mean square error of the probability of selecting the optimal strategy from the first round. The pre-convergence MSE is the MSE of the probability of selecting the optimal strategy accumulated from the first round to the round before the convergence. After convergence, MSE is the MSE of the probability of selecting the optimal strategy from the start of the 3000th round to the end of the experiment. The cumulative mean square error can effectively analyze the convergence of the strategy and the stability of the algorithm.

According to Figure 10 and Table 6, it can be concluded that the convergence time of the classic reinforcement learning algorithm under AM is 2–5 time faster than the processing rate of the IM framework. The AM framework algorithm can quickly find the optimal anti-jamming countermeasures in the single-round processing time period after convergence, which is 4–10 times faster than the IM framework with the same algorithm processing speed. From the analysis of MSE, QL, DQN, DDQN and DQN+LSTM have greater error oscillations before convergence under AM than under IM, but the oscillations of errors after convergence are all smaller tan IM.

Comprehensive analysis, compared to IM, the AM model proposed in this paper is more suitable for radar-jammer system. It has better convergence and higher processing efficiency.

## 5. Conclusions

This paper analyzes and studies the modern radar-jammer system, using reinforcement learning (RL) algorithms to improve the frequency-agile radar anti-jamming decision-making effect. The analysis shows that RL algorithms are suitable for the environment of radar-jammer system, but frequency-agile radar system has two characteristics of environmental instability and step changes in decision-making actions, which does not conform the complete Markov decision theory. As a result, the existing classical RL algorithms as QL [18], DQN [21], DDQN [24] and DQN+LSTM [25], which are applied to this scenario, have problems, e.g., low training efficiency and large error oscillation. For the above two problems, this paper proposes an efficient RL anti-jamming model for frequency-agile radar: transforming the radar-jammer detection model into a model that satisfies the MG theory, and is suitable for step change data. Experimental results show that the proposed model can improve the anti-jamming performance and efficiency of frequency-agile radar.

Based on the current work, further research will be carried out. First, the existing model have good experimental results for the finite radar state. When the radar state is infinite, the model needs to be further verified and optimized. Second, it is necessary to further verify the universality of the model and study whether the new RL algorithm is also applicable to the model framework proposed in this article.

## Figures and Tables

**Table 1:** Parameter definition of RL model based on single radar system.

RL Parameters	Radar System Parameters
Agent	Radar.
Environment	Target environment.
State	The status of the detected target $s_{t}^{RDR}$, which contains target speed, angle and distance information.
Action	Frequency agile anti-jamming cover pulse and tracking signal source emitted by radar $a_{t}^{RDR}$, which contains radar amplitude *A*, pulse width τ and pulse repetition period (PRF) β.
Reward	Obtain target information accuracy evaluation $r_{t}^{RDR}$.

**Table 2:** Definition of radar parameters of radar-jammer system environment model.

RL Parameters	Radar System Radar Side’s Parameters
Agent	Radar.
Environment	Target and jamming radar environment.
State	Status of detected targets with interference information $s_{t}^{RDR}$, which contains target speed υ, angle θ and distance *d* information, target error rate $\rho_{d}$.
Action	Frequency agile anti-jamming cover pulse and tracking signal source emitted by radar $a_{t}^{RDR}$, which contains radar amplitude *A*, pulse width τ and pulse repetition period (PRF) β.
Reward	Obtain target information accuracy assessment and whether the jamming signal locks the cover pulse $r_{t}^{RDR}$.

**Table 3:** Radar and jammer matrix game example.

	$a_{1}^{JAM}$	$a_{2}^{JAM}$
** $a_{1}^{RDR}$ **	(1,1)	(1,2)
** $a_{2}^{RDR}$ **	(2,1)	(2,2)

**Table 4:** Radar status description.

$s_{0}$	Radar initial state, which is the first radar echo signal, is described by target error rate $\rho_{d}$, $\rho_{d}\geq threshold$.
$s_{1}$–$s_{6}$	Radar intermediate state, $\rho_{d}\geq threshold$.
$s_{7}$	Radar final state, $\rho_{d}<threshold$.

**Table 5:** The reward value of the radar side and the jammer.

Target Error Rate	Radar’s Rewards	Jammer’s Reward
$\rho_{d}<5\%$	+5	−4
$5\%\leq \rho_{d}<15\%$	+4	−2
$15\%\leq \rho_{d}<35\%$	+1	−1
others	−4	+3

**Table 6:** Experimental comparison of processing time of classic RL algorithms under IM and AM.

Mode	Convergence Time (Time/μs)	Single-Round Decision Time After Convergence (Time/μs)	6000 Rounds Total Running Time (Time/μs)	MSE	
				Before Convergence	After Convergence
IM_QL [18]	101.576±0.12%	12.792±0.88%	837,581.728 ±0.69%	6.502±1.10%	1.901±2.97%
AM_QL	24.971±0.33%	1.554±4.01%	18,122.910 ±1.78%	7.151±0.84%	1.411±5.72%
IM_DQN [21]	210.774±1.04%	21.552±5.19%	1,083,521.021 ±0.72%	8.694±0.23%	1.907±1.41%
AM_DQN	117.863±0.42%	2.016±0.82%	159,102.375 ±0.38%	8.967±2.25%	1.433±2.16%
IM_DDQN [24]	191.861±0.23%	19.613±0.97%	106,707.731 ±3.61%	8.631±3.14%	2.072±0.55%
AM_DDQN	89.465±0.52%	1.976±2.49%	9153.983±1.08%	8.985±4.52%	1.361±4.62%
IM_DDQN+LSTM [25]	285.373±2.63%	30.184±0.12%	2,550,491.971 ±0.85%	6.781±1.70%	1.923±5.14%
AM_DQN+LSTM	132.926±7.01%	7.622±4.53%	283,561.026 ±2.37%	8.012±2.92%	1.876±3.85%

## Data Availability

Not applicable.
